# Pregnant women’s knowledge, attitudes, and practices on food safety: a cross-sectional study

**DOI:** 10.3389/fpubh.2026.1724884

**Published:** 2026-02-09

**Authors:** Merve Varol, Esma Aksoy Kendilci

**Affiliations:** 1 Institute of Graduate Education, Department of Food Safety (Interdisciplinary), Bitlis Eren University, Bitlis, Türkiye; 2 Department of Nutrition and Dietetics, Faculty of Health Sciences, Bitlis Eren University, Bitlis, Türkiye

**Keywords:** attitude, food safety, hygiene, practice, pregnancy

## Abstract

**Background:**

Pregnant women are physiologically more vulnerable to foodborne diseases because immune, hormonal, and metabolic changes reduce resistance to pathogens and increase the severity of dehydration and its fetal consequences. This study assessed the knowledge, attitudes and practices (KAP) of pregnant women regarding food safety.

**Methods:**

A cross-sectional study was conducted among 216 pregnant women attending Family Health Centers in Bitlis, Türkiye. Data were collected using a structured and validated questionnaire consisting of sections on socio-demographic characteristics, pregnancy-related factors, food safety knowledge (20 items), attitudes (18 items), and practices (11 items). Statistical analyses included descriptive statistics, chi-square tests and non-parametric group comparisons.

**Results:**

While general hygiene awareness was high (e.g., washing fruits/vegetables: 98.6%), knowledge of specific pathogens *Salmonella* (15.1%), *Listeria* (3.4%), *Toxoplasma gondii* (3.9%) was limited, which is expected as these pathogens are typically unfamiliar to the general public. Risky behaviors such as thawing frozen foods at room temperature (39.5%) and consuming unpasteurized milk were common. Misconceptions about packaged foods and food additives were striking (Approximately 95% perceived them as harmful), despite evidence showing that risks depend on additive type and dose rather than their mere presence. The mean attitude score was high (50.36 ± 4.63), and Higher educational level was significantly associated with safer practices such as checking expiry dates, refrigerating foods promptly, and using paper towels for hand drying (*p* = 0.002; *p* < 0.001; *p* < 0.001).

**Conclusion:**

Although pregnant women demonstrated positive attitudes and adequate general hygiene knowledge, gaps in pathogen-specific knowledge and several unsafe household practices persist. Antenatal education should prioritize clarifying true risk factors, correcting widespread misinformation, and reinforcing practical skills to promote safer food behaviors during pregnancy.

## 1 Introduction

Maternal nutrition and food safety are critical determinants of fetal growth, development, and pregnancy outcomes. While adequate and balanced nutrition is essential, the safety of consumed foods is equally important to prevent foodborne infections that may compromise maternal and fetal health. Food safety encompasses all stages from production to consumption; any disruption in this chain can increase the risk of contamination and adversely affect public health (1, 2).

Pregnancy is a physiologically vulnerable period marked by immunological and metabolic changes that heighten susceptibility to foodborne pathogens such as *Listeria monocytogenes*, *Toxoplasma gondii*, and *Salmonella* spp. According to the Centers for Disease Control and Prevention (CDC), pregnant women are nearly ten times more likely to develop *listeriosis* than non-pregnant adults (3). *Listeriosis* and similar infections often manifest as meningitis or septicemia, particularly among high-risk groups including pregnant women, infants, the older adults, and immunocompromised individuals (4).

Beyond pathogen-specific complications such as miscarriage or stillbirth, foodborne diseases may induce acute diarrheal episodes leading to dehydration and electrolyte imbalance. Pregnant women have increased physiological fluid requirements and reduced tolerance to dehydration, which may impair uteroplacental perfusion and negatively affect fetal outcomes. Thus, diarrheal dehydration represents an important but often overlooked mechanism linking food safety lapses to adverse maternal-fetal health consequences (5, 6).

Unsafe food handling practices can also trigger enteric infections that initiate the well-established diarrhea-malnutrition-infection vicious cycle. Each diarrheal episode exacerbates nutrient malabsorption and weakens immune function, thereby increasing vulnerability to subsequent infections (7–9). Undernutrition and impaired immunity, in turn, amplify the severity of infections, creating a self-reinforcing cycle particularly detrimental during pregnancy (10).

Globally, foodborne infections affect approximately 600 million individuals each year and account for 420,000 deaths, disproportionately impacting pregnant women and their fetuses (1). Although previous studies have shown that pregnant women often possess moderate to high levels of food-safety knowledge, unsafe behaviors remain common. For example, a study in Jordan reported that 65% of pregnant women had moderate knowledge yet continued unsafe handling practices (11), while Jevšnik et al. (12) similarly observed a gap between knowledge and practice among Slovenian pregnant women.

Despite global recognition of the importance of maternal food safety, research in Türkiye remains limited, and available studies provide only partial insight into pregnant women’s food-safety behaviors (13). Türkiye’s unique sociocultural and dietary characteristics underscore the need for updated, context-specific data addressing this gap.

The Knowledge-Attitude-Practice (KAP) model is widely used to understand food-safety behaviors. Knowledge forms the basis for awareness, attitudes reflect motivation, and practices represent the behavioral expression of this knowledge. This framework helps explain why adequate knowledge among pregnant women may not always translate into safe food-handling behaviors.

Therefore, the present study aimed to assess pregnant women’s knowledge, attitudes, and practices regarding food safety and to examine the associations between KAP outcomes and socio-demographic characteristics. This approach provides a comprehensive understanding of the factors influencing food-safety behavior during pregnancy and highlights areas requiring targeted public-health interventions.

## 2 Materials and methods

### 2.1 Study design and setting

This cross-sectional study was conducted between January and December across 10 Family Health Centers affiliated with the Central Community Health Center in Bitlis, Türkiye. This site was selected purposively because it is the primary antenatal follow-up center in the province, providing services to a socio-demographically diverse population of pregnant women. The center’s high patient volume and catchment area ensured accessibility and adequate representation of the target population. This study was designed and reported following the STROBE (Strengthening the Reporting of Observational Studies in Epidemiology) guidelines to ensure transparency, methodological rigor, and completeness of reporting.

### 2.2 Sample size determination

The study population consisted of 554 pregnant women. The minimum required sample size was calculated using the single-population proportion formula recommended by the World Health Organization for cross-sectional studies:


$$n=\frac{Z^{2}\cdot p\cdot (1-p)}{d^{2}}$$

Z = 1.96 (95% confidence level),

p = expected prevalence,

d = margin of error (0.05) (14).

A reference prevalence of 45.5% regarding food-safety knowledge among pregnant women was adopted from a recent study conducted (15). Thus, the minimum required sample size was 215. A total of 216 pregnant women were ultimately included in the study. Since one additional participant who met the inclusion criteria agreed to participate, the final sample became 216.

### 2.3 Sampling method

This study was conducted between January and December 2023 among pregnant women across 10 Family Health Centers affiliated with the Central Community Health Center the Central Family Health Centre in Bitlis, Türkiye. The study sample was selected using a probability based simple random sampling method among eligible pregnant women who presented to the Central Family Health Center during the data collection period. Day during data collection, all women presenting for routine antenatal care were screened for eligibility and invited to participate.

### 2.4 Participants: inclusion and exclusion criteria

Inclusion criteria:

Participants were eligible if they:

1) were currently pregnant,
2) were registered and followed at the Family Health Center,
3) were able to communicate in Turkish,
4) provided written informed consent.

Exclusion criteria:

Participants were excluded if they:

1) had cognitive, mental, or communication impairments that could compromise reliable responses,
2) presented with an acute obstetric complication requiring urgent medical intervention on the day of recruitment.

### 2.5 Questionnaire development and validation

The data were collected using a structured questionnaire composed of four sections:

(1) socio-demographic characteristics (10 items),
(2) pregnancy and health-related questions (4 items),
(3) food safety knowledge (20 items),
(4) food safety practices (11 items), and.
(5) food safety attitude (18 items).

The questionnaire items were adapted and developed based on validated instruments used in previous studies (11, 12, 15, 16).

A pilot test was conducted with 30 pregnant women to evaluate clarity, readability, and cultural appropriateness. Minor modifications in wording and item order were made based on participant feedback.

Content validity was assessed by a panel of five experts in nutrition, maternal health, and public health. The Content Validity Index (CVI) for the final questionnaire was 0.91, indicating excellent expert agreement.

### 2.6 Measurement and scoring of knowledge, practice and attitude (KAP)

#### 2.6.1 Knowledge assessment

Food safety knowledge was assessed through 20 items covering high-risk foods, foodborne pathogens, cross-contamination, refrigeration, and safe food-handling behaviors (12).

#### 2.6.2 Practice assessment

Food safety practice was evaluated through 11 items addressing behaviors related to purchasing (checking expiry dates), storing (refrigerator temperature), cooking, reheating, and personal hygiene. Responses were recorded on a three-point scale (“always,” “sometimes,” “never”) (16).

#### 2.6.3 Attitude assessment

The food safety attitude scale developed by Memiş (16) was used to assess participants’ attitudes. The original instrument consisted of 18 items rated on a 3-point Likert scale (“disagree,” “partially agree,” “agree”), with total possible scores ranging from 18 to 54. In this study, attitude scores were classified as follows:

- ≤27 = negative attitude,
- 28–44 = partially positive,
- ≥45 = positive attitude (16).

The Food Safety Attitude Scale developed by Memiş (16) reflects two conceptual dimensions based on the structure and content of the items. The first dimension, Caring About, consists of nine items (Items 1–9) that assess the extent to which individuals prioritize food hygiene during food selection, preparation, and consumption. These items reflect attitudes that tend to downplay the importance of hygiene, such as prioritizing taste, appearance, or satiety over hygienic considerations and disregarding hygiene-related warnings.

The second dimension, Internalization, also includes nine items (Items 10–18) and represents the degree to which food hygiene principles are internalized in daily life. This dimension captures positive attitudes toward food safety, including valuing hygiene as a primary criterion in food consumption, actively monitoring hygienic conditions, participating in hygiene-related activities, and considering hygiene regardless of circumstances.

In the present study, both the total food safety attitude score and the subdimension scores (Caring About and Internalization) were calculated and included in the analyses. Higher scores indicate more positive attitudes toward food safety.

The internal consistency of the Food Safety Attitude Scale was assessed using Cronbach’s alpha coefficients. The Caring About subdimension demonstrated good reliability with a Cronbach’s alpha of 0.83, while the Internalization subdimension showed acceptable reliability with a Cronbach’s alpha of 0.78. The overall scale exhibited high internal consistency, with a Cronbach’s alpha coefficient of 0.85. The Cronbach’s alpha for the Food Safety Attitude Scale in the current study was 0.86, indicating high internal consistency. The reliability coefficients for the subdimensions were also high, with a Cronbach’s alpha of 0.929 for the Caring About subscale and 0.834 for the Internalization subscale.

### 2.7 Variables

The dependent variables of this study were pregnant women’s food safety attitudes, knowledge, and behaviors. Food safety attitudes were assessed using the total Food Safety Attitude Scale score and the scores of its two subdimensions: Caring About and Internalization. Higher scores indicated more positive attitudes toward food safety.

Food safety knowledge (KAP-Knowledge) was evaluated using responses to questions related to foodborne pathogens, high-risk foods during pregnancy, and basic food safety principles. Food safety behaviors (KAP-Practice) were assessed based on self-reported practices concerning food purchasing, preparation, cooking, storage, and consumption.

The independent variables included selected socio-demographic characteristics, namely educational level, profession (employment status), and family income status. These variables were examined to determine their associations with food safety knowledge, attitudes (total and subdimension scores), and behaviors.

### 2.8 Statistical analysis

All data were analyzed using IBM SPSS Statistics version 29 (IBM Corp., Armonk, NY, USA). The normality of the numerical variables was assessed using the Kolmogorov–Smirnov test, which indicated that the data did not follow a normal distribution (*p* < 0.05). Accordingly, categorical variables were summarized as frequencies and percentages, while numerical variables were presented as mean, standard deviation, median, interquartile range, minimum, and maximum values.

Knowledge and practice variables were treated as categorical variables. Attitude was analyzed both as a continuous variable using the total attitude score and as a categorical variable. Categorization of attitude scores was performed according to the cut-off values recommended by the scale developer.

Because the study aimed to examine whether knowledge, attitude, and practice (KAP) outcomes differed across naturally occurring socio-demographic groups, and the data were non-normally distributed, non-parametric tests were applied. Mann–Whitney U test was used for comparisons between two independent groups. Kruskal-Wallis H test was used when comparing more than two groups. Associations between categorical variables were examined using the Chi-square test, and Fisher’s Exact test was applied when expected cell counts were below 5. A significance level of *p* < 0.05 was considered statistically meaningful for all analyses.

### 2.9 Ethical considerations

The study was approved by the Ethics Committee of Bitlis Eren University (Approval No: 2024/02–15; E.5205) and by the Provincial Directorate of Health under the Bitlis Governorship (E-39320161-602.01.0.-234398851). Informed consent was obtained from all participants prior to data collection. Participation was voluntary, and all participants were informed about the aim, process, and confidentiality of the study before providing written consent. The researcher collected the data from volunteer subjects through face-to-face interview technique. All procedures were conducted in accordance with the Declaration of Helsinki.

## 3 Results

Table 1 presents the socio-demographic characteristics of the participants. The mean age of the pregnant women was 28.56 ± 4.64 years. Among all participants, those with a bachelor’s degree or higher constituted the largest group (39.3%). Most women (97.2%) resided in the city center, and 86.1% lived in nuclear families. Regarding housing conditions, 79.6% lived in apartments, and 82.8% reported having a separate kitchen. More than half of the women (58.9%) were housewives. The mean gestational week was 21.68 ± 4.73, the mean gravidity was 1.79 ± 0.98, and the mean number of living children was 1.61 ± 0.86.

**Table 1:** Socio-demographic characteristics of the pregnant women (*n* = 216).

Variable	Category	*n*	%
Educational status	Primary School and below	52	24.1
	High School	79	36.6
	Bachelor’s degree or above	85	39.3
Place of residence	City centre	210	97.2
	District centre	4	1.9
	Village/Town	2	0.9
Family type	Nuclear Family	186	86.1
	Extended Family	30	13.9
Nature of your residence	Apartment	172	79.6
	Qualified detached house	44	20.4
Location of the kitchen	A separate kitchen in the house	178	82.8
	Open kitchen in the living room	37	17.2
Profession	Housewife	126	58.8
	Civil servant	35	16.4
	Self-employed/ Worker	53	24.8
Age (years) (Mean± SD) (Med (IQR), Min-Max)	28.56 ± 4.64 (32 (5), 18–41)		
Age at Marriage (years) (Mean± SD) (Med (IQR), Min-Max)	23.84 ± 3.56 (24 (6), 16–33)		
Gestational Week (Mean± SD) (Med (IQR), Min-Max)	21.60 ± 6.73 (24 (11), 2–38)		
Number of Pregnancy (Mean ± SD) (Med (IQR), Min-Max)	1.79 ± 0.98 (2 (1), 1–6)		
Number of Living Children (Mean ± SD) (Med (IQR), Min-Max)	1.61 ± 0.86 (1 (6), 1–5)		

Med: median, IQR: interquartile range, SD.: standard deviation, Min: minimum, Max: maximum, n: number, %: percentage.

Table 2 presents the distribution of food safety knowledge items among pregnant women (KAP -Knowledge). Physicians were the primary source of food safety information during pregnancy (74.9%), followed by dietitians (24.1%), media (20.4%), family older adults (17.6%), and midwives (13.0%). Most participants (62.5%) stated that high-risk foods (such as meat and cheese) should be purchased at the end of grocery shopping. A high proportion reported that hands should be properly washed before food preparation (85.2%), and almost all women (98.6%) considered washing fruits and vegetables before consumption to be important.

**Table 2:** Distribution of food safety knowledge items among pregnant women (KAP - Knowledge, *n* = 216).

Variable	Category	*n*	%
Who informed you about food safety during pregnancy? *	Physicians	161	75.9
	Dietitians	52	24.1
	Media (TV, radio, magazines, etc.)	47	20.4
	Family older adults	38	17.6
	Midwives	28	13.0
	Retailers’ recommendations when shopping	3	1.4
When should we buy risky foods (meat, cheese, etc.) when shopping for groceries?	At the beginning of shopping	81	37.5
	At the end of shopping	135	62.5
How do we wash our hands correctly and effectively before we start preparing food?	Water and soap	28	13.0
	Water only	4	1.9
	Water, soap and scrubbing between the fingers and around the wrists	184	85.1
Is it important to wash fruit and vegetables before eating them?	Yes	213	98.6
	No	2	0.9
	I do not know	1	0.5
Do you buy raw milk sold openly without packaging?	Yes	144	66.7
	No	72	33.3
How many minutes do you boil your raw milk at home? (minutes)	5–10	31	21.8
	10–15	58	40.8
	15 and above	53	37.3
Where is the best place to buy safe milk?	Sterilized and/or pasteurized boxed milk in shops	19	13.3
	Raw milk from the farm, i.e., directly from the producer	124	86.7
What are the points you pay attention to when consuming cheese during pregnancy? *	I avoid mouldy cheeses.	85	39.4
	I make sure that cheeses are produced from pasteurized milk.	76	35.2
	I do not buy cheeses sold open.	56	25.9
	I consume village cheese.	70	32.4
	I do not consume cheese produced from raw milk.	7	3.2
	Other (not consuming cheese)	2	0.9
Which of these foods spoils most quickly at room temperature?	Milk and dairy products	210	97.2
	Fruits and vegetables	4	1.9
	Cereals	2	0.9

*Multiple responses were given.

Regarding milk consumption, 66.7% reported consuming raw milk sold openly without packaging, and among them, 40.8% boiled it for 10–15 min. The most preferred source of “safe milk” was raw milk obtained directly from farms (86.7%). In terms of cheese consumption habits, avoiding moldy cheeses (39.4%) and choosing pasteurized products (35.2%) were commonly reported. Additionally, nearly all participants (97.2%) indicated that milk and dairy products were the most perishable foods at room temperature.

Table 3 summarizes pregnant women’s responses regarding thawing methods and the perceived safety of certain high-risk foods (KAP-Knowledge). A considerable proportion of the participants preferred to thaw frozen foods on the kitchen counter (39.5%). Regarding meat handling, 84.6% of the women believed that it is safe to refreeze thawed meat. Awareness of high-risk foods during pregnancy was generally high; the majority reported that consuming raw meat and raw meat products (98.1%), foods containing raw eggs (90.7%), undercooked eggs (88.4%), and raw milk (97.7%) is unsafe. Nearly all participants also stated that cheese made from raw milk (96.3%) and cold sandwiches or delicatessen meats (98.6%) are not appropriate during pregnancy. Additionally, most women indicated that rare or medium-cooked meats (95.4%) and raw sprouts (89.4%) are also unsafe to consume.

**Table 3:** Distribution of the responses of the pregnant women to some questions about thawing frozen foods and some potentially risky foods (KAP-Knowledge, *n* = 216).

Variable		Category	*n*	%
What is the best way to thaw frozen food?		On the kitchen counter, at room temperature	85	39.5
		In the microwave	17	7.9
		In the refrigerator	71	33.0
		In hot water	38	17.7
		Instant heat treatment	4	1.9
Is it safe to refreeze thawed meat?		Yes	181	84.6
		No	8	3.7
		I do not know	25	11.7
Is the following safe to eat during pregnancy?	Raw meat and foods prepared from raw meat (raw meatballs, sushi, etc.)	Yes	4	1.9
		No	212	98.1
	Pastries that contain raw eggs (homemade mayonnaise, mosaic cake, cookies, and wet cake)	Yes	20	9.3
		No	196	90.7
	Undercooked eggs (soft-boiled, easy, apricot-like eggs)	Yes	25	11.6
		No	191	88.4
	Raw milk (unpasteurised)	Yes	5	2.3
		No	211	97.7
	Cheese produced from raw milk	Yes	8	3.7
		No	208	96.3
	Cold sandwiches (containing ham, salami, smoked meat, smoked fish, smoked turkey)	Yes	3	1.4
		No	213	98.6
	Rare-medium cooked meats	Yes	10	4.6
		No	206	95.4
	Alfalfa or other raw sprouts	Yes	23	10.6
		No	193	89.4

According to Table 4, the distribution of pregnant women’s responses to selected food safety questions shows several notable patterns (KAP-Knowledge). Nearly half of the participants (46.3%) believed that herbal supplements such as ginger, chamomile, and mulberry leaves could be beneficial to use before taking medication during pregnancy. Ginger (60.5%) and chamomile (28.7%) were the most commonly preferred herbal supplements.

**Table 4:** Distribution of the responses of the pregnant women to some questions about food safety (KAP-Knowledge, *n* = 216).

Variable		Category	*n*	%
Is it better to benefit from herbal supplements (ginger, chamomile, mulberry leaf) rather than medication during pregnancy?		Yes	100	46.3
		No	78	36.1
		I do not know	38	17.6
Which of the herbal teas are safe during pregnancy? *		Chamomile	48	28.7
		Senna	2	1.2
		Mulberry leaf	16	9.6
		Ginger	101	60.5
Can we easily consume all seafood during pregnancy to intake omega-3 fatty acids?		Yes	50	23.3
		No	118	54.9
		I do not know	47	21.8
Which of the seafoods is harmful to consume during pregnancy?		Mussel	173	83.2
		Other(Sea bass, Trout, Horse mackerel)	35	16.8
Have you suffered from food poisoning before?		Yes	54	25.5
		No	158	74.5
Have you ever heard that these microorganisms are harmful to human health?	*Salmonella*	Yes	31	15.1
		No	174	84.9
	*Listeria*	Yes	7	3.4
		No	198	96.6
	*Toxoplasma gondii*	Yes	8	3.9
		No	198	96.1
	*Brucella*	Yes	95	45.2
		No	115	54.8
Do you believe that the consumption of packaged food during pregnancy is harmful?		Yes	199	92.6
		No	16	7.4
Do you believe that food additives are harmful during pregnancy?		Yes	208	96.7
		No	7	3.3

*Multiple responses were given.

More than half of the participants (54.9%) expressed negative views regarding the consumption of seafood for omega-3 intake, and a large proportion (83.2%) considered mussels particularly harmful. Additionally, 25.5% of the women reported having previously experienced food poisoning. Awareness of specific foodborne pathogens was very limited a result that is expected, as microorganisms such as *Listeria, Toxoplasma gondii*, or *Brucella* are typically unfamiliar to the general public. In contrast, nearly all participants viewed packaged foods (92.6%) and food additives (96.7%) as harmful, reflecting a lack of accurate information about the actual risks and acceptable uses of food additives during pregnancy.

Table 5 presents the distribution of pregnant women’s food safety practices according to educational status (KAP-Practice). Handwashing before meals was reported at very high levels across all education groups (96.2–98.8%). In contrast, drying hands with paper towels or napkins increased notably with higher education; the proportion of participants who reported “always/often” using this method was 55.8% in the primary school group, 68.4% in the high school group, and 86.9% among those with a bachelor’s degree or higher (*p* < 0.001). Contact with raw animal products an important behavior that increases the risk of cross-contamination decreased as education level increased; frequent contact was reported by 72.5% of the primary school group, 54.5% of the high school group, and 42.9% of the bachelor’s group (*p* = 0.005).

**Table 5:** Distribution of the responses of the pregnant women to behavioral practices related to food safety according to their educational status (KAP-Practice, *n* = 216).

Practices	Frequency category	Educational status						*p*	χ^2^
		Primary school and below (*n* = 52)		High school (*n* = 79)		Bachelor’s degree or above (*n* = 84)			
		*n*	%	*n*	%	*n*	%		
1. I wash my hands before meals.	Always/Quite Often	50	96.2	78	98.7	83	98.8	€	
	Frequently	2	3.8	1	1.3	1	1.2		
	Rarely/Never	0	0.0	0	0.0	0	0.0		
2. I use paper towels or tissues to dry my hands.	Always/Quite Often	29	55.8	54	68.4	73	86.9 ^a^	<0.001*	21.842
	Frequently	6	11.5	3	3.8	6	7.1		
	Rarely/Never	17	32.7	22	27.8	5	6.0		
3. I touch raw animal products.	Always/Quite Often	37	72.5	42	54.5	36	42.9	0.005*	15.089
	Frequently	5	9.8	18	23.4	15	17.9		
	Rarely/Never	9	17.6	17	22.1	33	39.3		
4. I wash hands before touching ready-to-eat foods.	Always/Quite Often	51	98.1	75	94.9	82	97.6	€	
	Frequently	1	1.9	4	5.1	2	2.4		
	Rarely/Never	0	0.0	0	0.0	0	0.0		
5. I wash my hands after touching raw food.	Always/Quite Often	50	96.2	75	94.9	82	97.6	€	
	Frequently	1	1.9	2	2.5	2	2.4		
	Rarely/Never	1	1.9	2	2.5	0	0.0		
6. I keep raw and ready-to-eat foods separately in the fridge.	Always/Quite Often	48	92.3	74	93.7	81	96.4	€	
	Frequently	2	3.8	4	5.1	3	3.6		
	Rarely/Never	2	3.8	1	1.3	0	0.0		
7. I refrigerate raw/cooked foods within 2 h.	Always/Quite Often	36	69.2	47	59.5	73	86.9	€	
	Frequently	13	25.0%	31	39.2%	11	13.1		
	Rarely/Never	3	5.8%	1	1.3%	0	0.0		
8. I check expiry dates before consuming foods.	Always/Quite Often	28	53.8%	38	48.1%	58	69.9 ^a^	0.002*	16.518
	Frequently	11	21.2%	29	36.7%	21	25.3		
	Rarely/Never	13	25.0%	12	15.2%	4	4.8		
9. I taste foods to check spoilage.	Always/Quite Often	36	69.2%	38	48.1%	56	66.7	0.056*	9.206
	Frequently	11	21.2%	32	40.5%	19	22.6		
	Rarely/Never	5	9.6%	9	11.4%	9	10.7		
10. I consume raw eggs/raw egg foods.	Always/Quite Often	1	1.9%	4	5.1%	2	2.4	0.836**	1.450
	Frequently	1	1.9%	1	1.3%	1	1.2		
	Rarely/Never	50	96.2%	74	93.7%	81	96.4		
11. I consume raw meat/raw meat foods.	Always/Quite Often	1	1.9%	3	3.8%	2	2.4	€	
	Frequently	1	1.9%	0	0.0%	1	1.2		
	Rarely/Never	50	96.2%	76	96.2%	81	96.4		

χ^2^ = Chi-Square Test. * Pearson’s Chi-squared test; ** Fisher’s exact test was used when the observation value was below 5, € no analysis, bold significant *p*-values (*p* < 0.05), ^a^ The group that makes the difference.

Other behaviors directly related to cross-contamination showed a similar pattern across education groups. Washing hands before touching ready-to-eat foods was reported by 84.6% of the primary school group, 82.3% of the high school group, and 89.3% of those with higher education, while handwashing after contact with raw foods was very common in all groups (92.3, 92.4, and 97.6%, respectively). Separating raw and cooked foods in the refrigerator another key practice for preventing cross-contamination was also widely adopted, with rates of 92.3% in the primary school group, 87.3% in the high school group, and 92.9% in the bachelor’s group.

Storing raw or cooked foods in the refrigerator within 2 h was more common among those with higher education (86.9%), compared with the primary school (69.2%) and high school groups (59.5%). A clear educational gradient was observed for checking expiration dates; this behavior was performed regularly by 53.8% of the primary school group, 48.1% of the high school group, and 69.9% of participants with a bachelor’s degree (*p* = 0.002). Tasting.

food to check for spoilage was more prevalent among those with primary education (69.2%). Consumption of raw eggs, foods containing raw eggs, or raw meat was low across all education groups.

Table 6 shows the distribution of participants’ responses to food-safety knowledge items by educational status (KAP-Knowledge). Regarding the question of when risky foods should be purchased during grocery shopping, the proportion of participants who correctly indicated that such items should be bought at the end of the shopping process increased with higher educational levels. This rate was 40.4% among those with primary education, 49.4% among high school graduates, and 88.2% among participants with a bachelor’s degree (*p* < 0.001). The proportion of individuals who could correctly describe proper handwashing before food preparation namely the water-soap-scrubbing technique was similarly higher among those with more education, rising from 69.2% in the primary school group to 83.5% in the high school group and 96.5% in the bachelor’s degree group (*p* < 0.001). The importance of washing fruits and vegetables before consumption was reported at consistently high levels across all education groups.

**Table 6:** Distribution of participants’ responses to food-safety knowledge items according to their educational status (KAP-Knowledge).

Knowledge items	Category	Educational status						*p*	χ^2^
		Primary school and below (*n* = 52)		High school (*n* = 79)		Bachelor’s degree or above (*n* = 84)			
		*n*	%	*n*	%	*n*	%		
When should we buy risky foods (meat, cheese, etc.) when shopping for groceries?	At the beginning of shopping	31	59.6	40	50.6	10	11.8	<0.001*	40.684
	At the end of shopping	21	40.4	39	49.4	79	88.2^a^		
How do we wash our hands correctly and effectively before we start preparing food?	Water only or Water and soap	16	30.8	13	16.5	3	3.5	<0.001**	19.235
	Water, soap and scrubbing between the fingers and around the wrists	36	69.2	66	83,5	82	96.5 ^a^		
Is it important to wash fruit and vegetables before eating them?	Yes	50	96.2	78	98.7	85	100.0	€	
	No	1	1.9	1	1.3	0	0.0		
	I do not know	1	1.9	0	0.0	0	0.0		
Do you buy raw milk?	Yes	32	61.5	50	63.3	62	72.9	0.283*	2.526
	No	20	38.5	29	36.7	23	27.1		
How many minutes do you boil your raw milk at home? (minutes)	5–10	7	21.9	5	10.2	19	31.1	0.077*	8.422
	10–15	15	46.9	24	49.0	19	31.1		
	15 and above	10	31.2	20	40.8	23	37.7		
Where is the best place to buy safe milk?	Sterilized and/or pasteurized boxed milk in shops	0	0.0	2	4.0	17	27.4	€	
	Raw milk from the farm, i.e., directly from the producer	31	100.0	48	96.0	45	72.6		
Which of these foods spoils most quickly at room temperature?	Milk and dairy products	49	94.2	77	97.5	84	98.8	€	
	Fruits and vegetables	1	1.9	2	2.5	1	1.2		
	Cereals	2	3.8	0	0.0	0	0.0		

χ^2^ = Chi-Square Test.*Pearson Chi-Square Test; **Fisher’s Exact value was used when the observation value was below 5. ^a^The group that makes the difference, € no analysis, bold significant *p*-values (*p* < 0.05).

Purchasing raw milk sold openly did not differ significantly by educational status (*p* = 0.283). When asked about the boiling duration for openly purchased raw milk, the proportion of participants reporting that they boiled it for 10–15 min was 46.9, 49.0 and 31.1% in the primary, high school, and bachelor’s groups, respectively; this difference was not statistically significant (*p* = 0.077). Due to insufficient observations, statistical comparison for the preferred source of safe milk could not be performed; however, a higher preference for pasteurized milk was observed among those with a bachelor’s degree, while obtaining raw milk directly from a farm tended to be more common among participants with lower educational levels.

For the item assessing which food spoils most quickly at room temperature, almost all participants selected milk and dairy products, with rates ranging between 94.2 and 98.8% across groups.

Table 7 illustrates participants’ knowledge of safe thawing methods, refreezing practices, and foods that should be avoided during pregnancy, stratified according to their educational status (KAP-Knowledge). When preferences for thawing frozen foods were examined, thawing at room temperature was more common among participants with lower educational levels; the proportion was 46.2% in the primary school group and substantially higher than that observed among those with a bachelor’s degree or above. In contrast, the refrigerator recognized as a safer thawing method was used more frequently as education increased. The proportion selecting this method was significantly lower in the primary school (30.8%) and high school groups (23.1%) compared with those with a bachelor’s degree (43.5%). Although microwave thawing was reported infrequently across all groups, it was most common among participants with higher education (11.8%). Overall, these patterns indicate that risky thawing practices decrease and safer approaches become more prevalent as educational level rises (*p* = 0.020).

**Table 7:** Participants’ knowledge of safe thawing methods, refreezing practices, and foods that should be avoided during pregnancy, stratified according to their educational status (KAP-Knowledge).

Knowledge items		Category	Educational status						*p*	χ^2^
			Primary school and below (*n* = 52)		High school (*n* = 79)		Bachelor’s degree or above (*n* = 84)			
			*n*	%	*n*	%	*n*	%		
What is the best way to thaw frozen food?		On the kitchen counter, at room temperature	24	46.2	39	50.0	22	25.9 ^a^	0.020**	15.022
		In the microwave	3	5.8	4	5.1	10	11.8 ^a^		
		In the refrigerator	16	30.8	18	23.1	37	43.5^a^		
		In hot water	9	17.3	17	21.8	16	18.8 ^a^		
Is it safe to refreeze thawed meat?		Yes	44	86.3	55	70.5^a^	82	96.5	<0.001**	21.161
		No or I do not know	7	13.7	23	29.5	3	3.5		
Is the following safe to eat during pregnancy?	Raw meat and foods prepared from raw meat (raw meatballs, sushi, etc.)	Yes	2	3.8	1	1.3	1	1.2	0.555**	1.538
		No	50	96.2	78	98.7	84	98.8		
	Pastries that contain raw eggs (homemade mayonnaise, mosaic cake, cookies, and wet cake)	Yes	4	7.8	13	16.5^a^	3	3.5	0.016*	8.278
		No	47	92.2	66	83.5	82	96.5		
	Undercooked eggs (soft-boiled, easy, apricot-like eggs)	Yes	8	15.4	10	12.7	7	8.2	0.416*	1.754
		No	44	84.6	69	87.3	78	91.8		
	Raw milk (unpasteurised)	Yes	2	3.8	3	3.8	0	0.0	€	
		No	50	96.2	76	96.2	85	100.0		
	Cheese produced from raw milk	Yes	3	5.8	5	6.3	0	0.0	€	
		No	49	94.2	74	93.7	85	100.0		
	Cold sandwiches (containing ham, salami, smoked meat, smoked fish, smoked turkey)	Yes	2	3.8	1	1.3	0	0.0	€	
		No	50	96.2	78	98.7	85	100.0		
	Rare-medium cooked meats	Yes	3	5.8	5	6.3	2	2.4	0.389**	1.822
		No	49	94.2	74	93.7	83	97.6		
	Alfalfa or other raw sprouts	Yes	4	7.7	15	19.0^a^	4	4.7	0.009*	9.406
		No	48	92.3	64	81.0	81	95.3		

χ^2^ = Chi-Square Test.*Pearson Chi-Square Test; **Fisher’s Exact value was used when the observation value was below 5.^a^The group that makes the difference, € no analysis, bold significant *p*-values (*p* < 0.05).

A notable finding also emerged regarding misconceptions about refreezing thawed meat: the proportion of participants who believed refreezing to be safe was highest in the bachelor’s group (96.5%), followed by the primary school (86.3%) and high school groups (70.5%), and this difference was statistically significant (*p* < 0.001). Knowledge about avoiding raw meat and meat products during pregnancy was consistently high across all groups, with no significant differences detected. However, responses regarding pastries containing raw eggs varied by education, with high school participants more frequently considering such foods safe (16.5%) compared with the primary school (7.8%) and bachelor’s groups (3.5%) (*p* = 0.016). No significant differences were observed for undercooked eggs, raw milk, cheese made from unpasteurized milk, or cold sandwiches, and some items could not be analyzed due to low cell frequencies. Finally, a significant variation was noted for the consumption of raw sprouts, as 19.0% of high school participants considered them safe higher than in both the primary school (7.7%) and bachelor’s groups (4.7%) (*p* = 0.009).

Table 8 illustrates participants’ knowledge of safe thawing methods, refreezing practices, and foods that should be avoided during pregnancy, stratified according to their educational status (KAP-Knowledge). The belief that herbal supplements are safer than medication during pregnancy was most common among participants with a primary school education (61.5%), decreasing to 50.6% in the high school group and further to 32.9% among those with a bachelor’s degree (*p* = 0.003). The proportion of participants who believed that all types of seafood can be safely consumed during pregnancy to obtain omega-3 fatty acids was similar in the primary and high school groups (30.8 and 30.4%, respectively) but dropped markedly in the bachelor’s group (11.9%; *p* < 0.001). When asked which seafood is unsafe during pregnancy, the percentage identifying mussels as harmful increased substantially with education level, rising from 57.1% in the primary school group to 89.5% in the high school group and 92.8% among bachelor’s degree holders (*p* < 0.001). The proportion of participants who had previously experienced food poisoning also increased in parallel with education (%11.5 → %25.3 → %34.6; *p* = 0.012). Awareness of Salmonella as a harmful microorganism was very low among those with primary education (2.0%) but increased to 12.0% in the high school group and 26.6% in the bachelor’s group; similarly, knowledge of Brucella increased with education (29.4% → 41.0% → 59.3%; *p* = 0.002). Belief that packaged foods are harmful during pregnancy was consistently high across all groups (89.3–94.9%), showing no significant difference. In contrast, the belief that food additives pose risks during pregnancy increased slightly with education, from 90.4% in the primary school group to 98.7 and 98.8% in the high school and bachelor’s groups, respectively (*p* = 0.016).

**Table 8:** Participants’ knowledge of safe thawing methods, refreezing practices, and foods that should be avoided during pregnancy, stratified according to their educational status (KAP-Knowledge).

Knowledge items		Category	Educational status						*p*	χ^2^
			Primary school and below (*n* = 52)		High school (*n* = 79)		Bachelor’s degree or above (*n* = 84)			
			*n*	%	*n*	%	*n*	%		
Is it better to benefit from herbal supplements (ginger, chamomile, mulberry leaf) rather than medication during pregnancy?		Yes	32	61.5 ^a^	40	50.6	28	32.9	0.003*	15.819
		No	9	17.3	28	35.4	41	48.2		
		I do not know	11	21.2	11	13.9	16	18.8		
Can we easily consume all seafood during pregnancy to intake omega-3 fatty acids?		Yes	16	30.8	24	30.4	10	11.9	<0.001*	42.292
		No	19	36.5	30	38.0	69	82.1^a^		
		I do not know	17	32.7	25	31.6	5	6.0		
Which of the seafoods is harmful to consume during pregnancy?		Mussel	28	57.1^a^	68	89.5	77	92.8	<0.001*	31.342
		Other(Sea bass, Trout, Horse mackerel)	21	42.9	8	10.5	6	7.2		
Have you suffered from food poisoning before?		Yes	6	11.5	20	25.3	28	34.6	0.012*	8.849
		No	46	88.5	59	74.7	53	65.4		
Have you ever heard that these microorganisms are harmful to human health?	*Salmonella*	Yes	1	2.0	9	12.0	21	26.6^a^	<0.001*	15.536
		No	50	98.0	66	88.0	58	73.4 ^a^		
	*Listeria*	Yes	0	0.0	2	2.6	5	6.4	€	
		No	51	100.0	74	97.4	73	93.6		
	*Toxoplasma gondii*	Yes	2	3.9	2	2.6	4	5.1	0.899**	0.752
		No	49	96.1	75	97.4	74	94.6		
	*Brucella*	Yes	15	29.4	32	41.0	48	59.3^a^	0.002*	12.143
		No	36	70.6	46	59.0	33	40.7 ^a^		
Do you believe that the consumption of packaged food during pregnancy is harmful?		Yes	49	94.2	75	94.9	75	89.3	0.339*	2.166
		No	3	5.8	4	5.1	9	10.7		
Do you believe that food additives are harmful during pregnancy?		Yes	47	90.4	78	98.7	83	98.8 ^a^	0.016**	6.659
		No	5	9.6	1	1.3	1	1.2		

χ^2^ = Chi-Square Test. * Pearson Chi- Square Test; **Fisher’s Exact value was used when the observation value was below 5.^a^The group that makes the difference, € no analysis, bold significant *p*-values (*p* < 0.05).

Table 9 presents the distribution of food safety attitude levels among participants, providing descriptive context necessary for interpreting the subsequent relationship analyses (KAP-Attitude). The majority of participants (91.2%) demonstrated “highly positive” while 8.8% exhibited “partially positive” and a very small proportion showed negative attitudes. On the ‘caring about’ subscale, 96.3% of women displayed “highly positive” whereas only 1.9% presented negative attitudes. Similarly, in the ‘internalization’ subscale, 80.1% of participants showed a “highly positive” 17.6% reported a “partially positive” and 2.3% demonstrated a negative attitude. The mean and standard deviation value of the food safety attitude scale was 50.36 ± 4.63, and the lower and upper values were 29–54, respectively.

**Table 9:** Distribution of food safety attitudes among the pregnant women (KAP-Attitude).

Variable	Category	*n*	%
Food Safety Attitude Scale	Partially positive	19	8.8
	Highly positive	197	91.2
Caring about	Negative	4	1.9
	Partially positive	4	1.9
	Highly positive	208	96.3
Internalization	Negative	5	2.3
	Partially positive	38	17.6
	Highly positive	173	80.1
Caring about (Mean± SD) (Med (IQR), Min-Max)		26.21 ± 2.66 (27 (0, 0), 9–27)	
Internalization (Mean± SD) (Med (IQR), Min-Max)		24.14 ± 3.29 (25 (4), 10–27)	
Food safety attitude scale (Mean± SD) (Med (IQR), Min-Max)		50.36 ± 4.63 (52 (4), 29–54)	

Med: median, IQR: interquartile range, SD.: standard deviation, Min: minimum, Max: maximum, n: number, %: percentage.

Table 10 presents the comparison of pregnant women’s Food Safety Attitude Scale scores and subscale scores across different variables (KAP-Attitude). No statistically significant differences were observed in the overall attitude scale or its subscales based on educational status (*p* > 0.05). The mean scores of women with primary school education or below, high school education, and those with a bachelor’s degree or higher were similar.

**Table 10:** Comparison of the pregnant women’s scores of food safety attitude scale and their subscale scores according to different variables (KAP-Attitude, *n* = 216).

Variable	Category	Score of food safety attitude scale	Caring about subscale	Internalization subscale
		Mean ± SD	Mean ± SD	Mean ± SD
Educational status	Primary school and below	49.48 ± 6.38	25.75 ± 3.74	23.73 ± 4.14
	High school	50.06 ± 4.26	26.15 ± 2.39	23.91 ± 3.20
	Bachelor’s degree or above	51.16 ± 3.47	26.55 ± 2.02	24.61 ± 2.71
		H:3.370 p:0.185	H:2.848 p:0.241	H:2.017 p:0.365
Profession	(1) Housewife	50.13 ± 4.87	26.22 ± 2.52	23.90 ± 3.52
	(2) Civil Servant	49.66 ± 4.68	26.29 ± 3.05	23.37 ± 3.48
	(3) Self-employed/ worker	51.34 ± 3.92	26.11 ± 2.80	25.23 ± 2.22
		H:5.905 p:0.052	H:0.510 p:0.775	**H:9.698** p:0.008* **1 < 3, 2 < 3**
Income Status of the Family	Minimum wage and below	49.79 ± 5.43	25.89 ± 3.38	23.90 ± 3.55
	Above minimum wage	51.14 ± 3.05	26.67 ± 0.82	24.48 ± 2.86
		U:1.141 p:0.254	U:0.892 p:0.372	U:0.872 p:0.383

H = Kruskal Wallis Test, U = Mann Whitney Test. 1 < 3, 2 < 3: The group that makes the difference. Bold significant *p*-values (*p* < 0.05).

A statistically significant difference emerged in the internalization subscale across occupational groups (*p* = 0.008). The mean internalization score was 23.90 ± 3.52 among housewives, 23.37 ± 3.48 among civil servants, and 25.23 ± 2.22 among self-employed/worker participants. Post-hoc analysis indicated that the scores of housewives and civil servants were significantly lower than those of self-employed/worker women. No significant differences were found in the total attitude scale or the ‘caring about’ subscale across occupational categories (*p* > 0.05), and the mean scores of all groups were similar.

Similarly, no statistically significant differences were detected between the Food Safety Attitude Scale (or its subscales) and income levels (*p* > 0.05), with mean scores being comparable across income groups.

## 4 Discussion

This study examined food safety attitudes among pregnant women and identified key socio-demographic factors associated with these attitudes. The findings indicate that educational level, profession, and family income status are significantly related to food safety attitude scores, suggesting that socio-economic position plays an important role in shaping perceptions and concerns about food safety during pregnancy. These results underscore the importance of targeted public health strategies that consider social and educational inequalities when promoting food safety awareness among pregnant women.

Understanding food safety–related knowledge, attitudes, and practices among pregnant women is essential for preventing foodborne infections and protecting maternal fetal health. Consistent with previous studies, the present findings indicate that although most pregnant women possess moderate to high levels of food-safety knowledge, unsafe food-handling behaviors persist (11, 12, 17).

In this study, participants demonstrated strong awareness of general hygiene practices such as handwashing (85.1%) and washing fruits and vegetables (98.6%). However, several high-risk behaviors remained common. Notably, 39.5% reported thawing frozen foods at room temperature a practice known to promote rapid bacterial multiplication as the outer surface enters the 5–60 °C “danger zone” while the core remains frozen (18–20). Similarly, 66.7% consumed raw milk typically boiled for 10–15 min. Evidence shows that bringing raw milk to a full boil and maintaining it for 2–5 min is adequate to inactivate common pathogens such as Brucella, *Listeria monocytogenes*, Salmonella, and *E. coli* O157: H7 (21, 22). Prolonged boiling does not enhance microbiological safety; instead, it accelerates the loss of heat-sensitive nutrients including B vitamins and folate and may degrade protein quality (23, 24). These findings underscore the importance of educating pregnant women about safe and nutritionally optimal heat-treatment methods. In addition, international authorities consistently advise against consuming unregulated raw milk due to its high contamination risk (22, 25).

A substantial knowledge gap was observed regarding foodborne pathogens: only 15.1% recognized Salmonella, and only 3–4% identified Listeria or Toxoplasma gondii. Similar low awareness levels have been reported among pregnant women in the United Arab Emirates and Jordan (11, 15). Given the severe maternal and fetal complications associated with these pathogens, antenatal education should explicitly address pathogen-specific risks.

Although 91.2% of participants exhibited positive attitudes toward food safety consistent with studies from Slovenia and Bangladesh (12, 26) these attitudes did not consistently translate into safe practices. This “knowledge–behavior gap” has been widely documented (27, 28) and highlights the need for interventions that incorporate both information and practical skill-building components.

Educational level emerged as a significant predictor of both attitudes and behaviors, in line with findings from Ghana, Jordan, and the UAE (11, 15, 17). Higher-educated women were more likely to engage in safer practices such as checking expiry dates, timely refrigeration, and using paper towels for hand drying. Paper towels are microbiologically safer than cloth towels because they physically remove moisture and bacteria and do not promote cross-contamination; shared cloth towels can harbor *E. coli*, *Staphylococcus aureus*, and Salmonella (18, 29, 30). This may explain the more consistent adoption of safe drying methods among highly educated participants.

Environmental and contextual factors also influenced food safety compliance. Limited access to appropriate storage facilities, lower household income, and culturally rooted cooking practices can lead to behaviors such as inadequate refrigeration, unsafe thawing, and prolonged storage of perishable foods. These conditions are known to promote bacterial growth and increase contamination risk (18, 31, 32). Thus, improving food safety requires not only individual-level education but also structural and environmental support.

Misinformation regarding packaged foods and food additives was striking, with nearly 95% of participants perceiving these products as harmful. This pattern is consistent with findings from China, where more than 80% of pregnant women reported similar misconceptions (33). Although certain food additives particularly nitrites, nitrates, and some colorants have been associated with potential health effects such as metabolic disturbances, neurobehavioral symptoms, or increased cancer risk under high or prolonged exposure (34, 35), regulatory bodies emphasize that additives used within legal limits also provide important benefits, including improved food stability, reduced microbial risk, and enhanced shelf life (36). Therefore, the generalized belief that “all additives are harmful” reflects a knowledge gap that may divert attention away from truly high-risk practices (e.g., consuming unpasteurized milk, improper thawing). This underscores the need for clearer, evidence-based risk communication during antenatal counseling to help pregnant women distinguish scientifically substantiated risks from misconceptions.

The findings of this study should be interpreted in light of certain considerations regarding generalisability. As the study was conducted among pregnant women from a specific region in Türkiye using a cross-sectional design, the results may not be fully generalisable to all pregnant women nationwide or to different cultural and socio-economic contexts. However, the findings underscore the need for multi-level, sustained food safety interventions targeting pregnant women. Knowledge alone is insufficient; practical demonstrations, repeated reinforcement, culturally sensitive counseling, and supportive environmental conditions are essential. In this context, addressing socio-economic disparities and tailoring interventions according to educational level and income status may further enhance the effectiveness of food safety promotion efforts. As emphasized in recent literature (37, 38), long-term educational strategies delivered through routine antenatal care by physicians, dietitians, midwives, and public-health professionals may substantially improve food safety practices and enhance maternal-fetal protection in Türkiye.

## 5 Limitations and strengths

This study has several limitations that should be taken into account. First, the cross-sectional design precludes establishing causal relationships between knowledge, attitudes, and practices; only associations can be inferred. Second, although key socio-demographic variables were assessed, several potential confounders such as household food storage conditions, refrigerator temperature, kitchen facilities, cultural cooking habits, food affordability, general health literacy, and prior experience with foodborne illness were not measured. The absence of these factors may partly explain variations in food-safety behaviors. Third, although the questionnaire included items related to hygiene and safe storage, it did not explicitly assess cross-contamination behaviors (e.g., using separate cutting boards or cleaning utensils between raw and ready-to-eat foods). Similarly, the omission of “critical handwashing moments” (e.g., after handling raw meat or after using the toilet) may underestimate gaps in essential hygiene practices. Finally, the study was conducted in a single province, which may limit generalizability to regions with different cultural or environmental contexts.

Despite these limitations, the study has notable strengths. It provides one of the few recent assessments of food-safety KAP among pregnant women in Türkiye and addresses an identified gap in the national literature. The questionnaire demonstrated strong reliability and content validity, and the study offers practical insights for improving antenatal food-safety education and developing more targeted interventions.

## 6 Conclusion

This study highlights important gaps in pregnant women’s food-safety knowledge and behaviors in Türkiye. Although general hygiene awareness and attitudes toward food safety were strong, awareness of specific foodborne pathogens and adherence to certain critical practices such as safe thawing methods and appropriate handling of raw milk remained limited. Misconceptions regarding packaged foods and food additives were also widespread, indicating that inaccurate beliefs may overshadow actual high-risk behaviors. Education level was a key determinant of safer practices, underscoring the importance of targeted health education.

Strengthening antenatal counseling with clear, practical, and evidence-based food-safety guidance is essential to reduce preventable foodborne risks during pregnancy. Interventions should focus on pathogen-specific education, correction of common misconceptions, and reinforcement of safe household practices. A coordinated public-health approach that integrates education with supportive environmental and community resources may contribute significantly to protecting maternal and fetal health.

Future research should include key environmental and behavioral determinants such as food storage conditions, kitchen practices, and health literacy and use longitudinal or observational designs to clarify causal pathways. Additionally, intervention studies are needed to evaluate the effectiveness of targeted antenatal food-safety education programs.

## Data Availability

The datasets generated and/or analysed during the current study are not publicly available due to the protection of personal data and ethical restrictions, as participants were assured that their information would remain confidential and used solely for scientific purposes. Data may be made available from the corresponding author upon reasonable request and subject to collective participant consent.
